# Mesonephric‐Like Adenocarcinoma of the Endometrium and the Impact of the Updated 2023 FIGO Staging System

**DOI:** 10.1155/crog/9935815

**Published:** 2026-05-09

**Authors:** Joy Ogunmuyiwa, Alexandra Hamilton, Judy Hayek, Jocelyn Ogunmuyiwa, Constantine Gorelick, Daniel Levitan, Hani Ashamalla

**Affiliations:** ^1^ NewYork-Presbyterian Brooklyn Methodist Hospital, Brooklyn, New York, USA, nyp.org; ^2^ NewYork-Presbyterian Weill Cornell Medical Center, New York, USA, nyp.org; ^3^ University of Medicine and Health Sciences, Basseterre, Saint Kitts and Nevis, umhs-sk.org

## Abstract

Mesonephric adenocarcinomas are rare neoplasms derived from remnants of the paired mesonephric ducts. Neoplasms arising from the uterine corpus that are morphologically and immunohistochemically similar to true mesonephric carcinomas, but do not arise from mesonephric remnants, are termed “mesonephric‐like” adenocarcinomas. The diagnosis of mesonephric‐like adenocarcinoma is pathologically challenging. These tumors often exhibit a mixture of morphologic patterns of more common adenocarcinomas. Although their morphologic, immunohistochemical, and molecular profiles have been recently defined, much remains unknown about mesonephric‐like adenocarcinomas. A better understanding of mesonephric‐like andenocarcinoma’s pathogenesis and molecular classification is needed to improve diagnosis and guide management of this rare and aggressive subtype of endometrial cancer. An updated International Federation of Gynecology and Obstetrics (FIGO) staging system for endometrial carcinoma was introduced in June 2023, upstaging aggressive histological subtypes such as mesonephric‐like adenocarcinoma. In this report, we describe a patient with mesonephric‐like adenocarcinoma arising in the uterine corpus, highlight the clinical significance of 2023 FIGO restaging, and discuss the multidisciplinary approach to treatment.

## 1. Introduction

Mesonephric ducts, also known as Wolffian ducts, contribute to the development of male reproductive structures in males but are largely vestigial in females [1]. Despite the limited role of mesonephric ducts in females, neoplasms originating from true mesonephric remnants can occur. Mesonephric adenocarcinoma of the endometrium is a rare malignant tumor of the female genital tract comprising less than 1% of all gynecological malignancies [1, 2]. They typically arise in the wall of the uterine cervix or vagina. However, there have been some reported cases in the uterine corpus [3, 4]. Neoplasms arising in the uterine corpus that are morphologically and immunohistochemically similar to true mesonephric carcinomas but do not arise from mesonephric remnants are termed “mesonephric‐like” adenocarcinomas [2]. They tend to behave aggressively, with more than half of the published cases presented as an advanced stage (FIGO 2009 Staging ≥ II) at diagnosis [5]. They also demonstrate an increased risk of recurrence and early metastasis to the lungs and liver [5].

Due to its rarity and overlapping histological features with other uterine tumors, the diagnosis of mesonephric‐like adenocarcinoma can pose a challenge. Understanding its unique histopathological characteristics, molecular markers, and clinical implications is crucial for accurate diagnosis, prognostication, and effective management. In this report, we describe a case of mesonephric‐like adenocarcinoma arising in the uterine corpus and discuss the implications of the 2023 FIGO staging update for the management of aggressive endometrial cancer subtypes.

## 2. Case Presentation

A written informed consent was obtained from the patient. A gravida 1 para 0 61 year old female presented with a 1‐month history of postmenopausal bleeding in December 2023. An office pelvic ultrasound demonstrated a thickened, 2.3 cm endometrium. Endometrial biopsy was completed. Histologically, the tumor was composed of malignant glands, which showed positivity for PAX8, TTF1, GATA3, CK7, and CD10 (luminal staining pattern) via immunohistochemical analysis. Estrogen receptor (ER) was negative and progesterone peceptor (PR) < 1%. p53 demonstrated normal expression (wildtype), and p16 was negative. Mismatch repair (MMR) markers showed intact nuclear expression (MMR‐proficient phenotype). The presumptive diagnosis was mesonephric‐like adenocarcinoma.

Preoperative imaging was significant for a 2.6 × 5.4 × 3.6 cm endometrial mass located in the left posterior fundus, a leiomyomatous uterus, a thickened endometrial stripe, and prominent bilateral iliac lymph nodes, but negative for any evidence of metastatic disease (Figure 1). The serum tumor marker, CA125, was slightly elevated at 73 U/mL. She underwent a total robotic hysterectomy, bilateral salpingo‐oophorectomy, bilateral pelvic sentinel lymph node dissection, and omentectomy.

Figure 1T2‐weighted MRI demonstrates a leiomyomatous uterus with a 2.6 × 5.4 × 3.6 cm endometrial mass located in the left posterior fundus/body (arrow), shown in (A) sagittal and (B) coronal planes.

**Figure:**
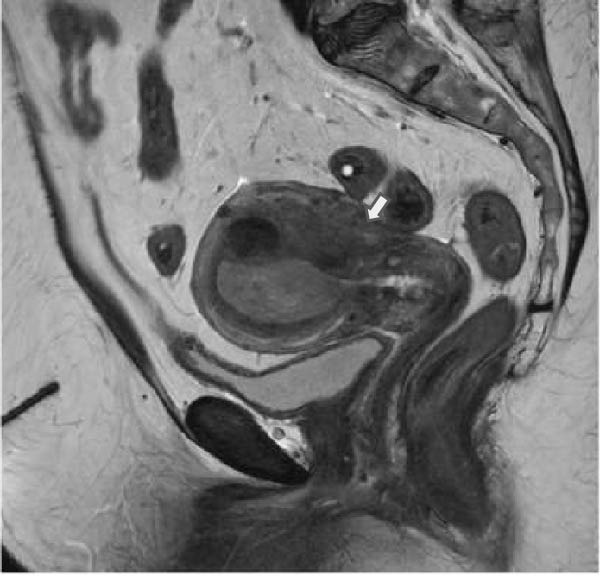
(A)

**Figure:**
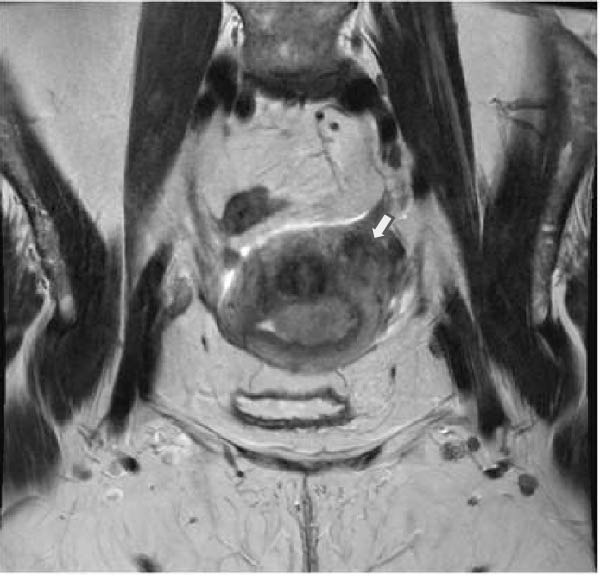
(B)

### 2.1. Final Pathology

**Figure 2:**
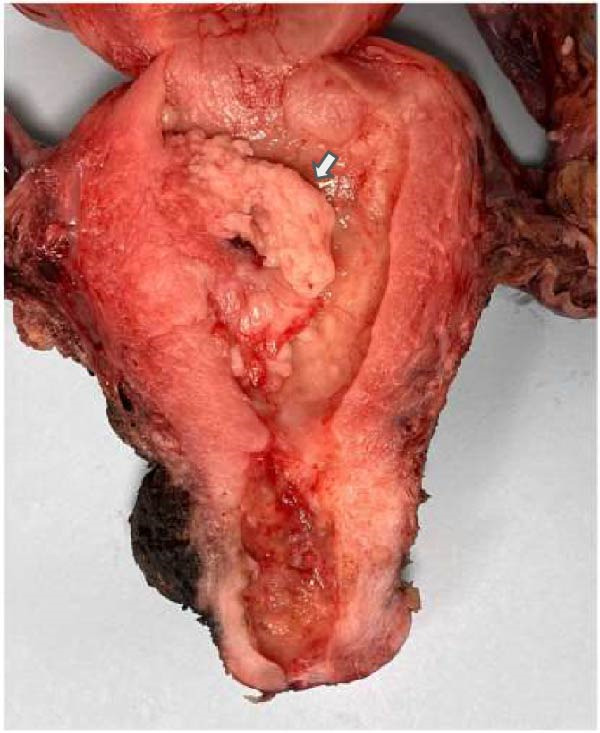
Gross findings of mesonephric‐like adenocarcinoma of the uterine corpus (posterior view). The tumor is bulky, lobulated, and pink‐tan to tan‐white with a prominent exophytic component extending through the cervical os.

The gross description of the tumor was bulky with a prominent exophytic component extending through the cervical os (Figure 2). There was myometrial invasion, estimated to be less than 50%. There was extensive lymphovascular invasion, defined as greater than or equal to 5 vessel involvement. There was no involvement of the uterine serosa, lower uterine segment, or cervical stroma. All regional lymph nodes were negative for tumor cells.

Microscopic examination revealed a tumor composed of irregular glands, punctuated by foci of microacinar‐to‐glandular architecture (Figure 3). Tumor cells were arguably more basaloid and contained less cytoplasm than a typical endometrioid carcinoma. The tumor was positive for PAX8 (diffuse), TTF1 (patchy), GATA3 (focal/patchy), CK7 (patchy), and CD10 (luminal staining), while negative for ER, PR, p16, and CK20. There was normal p53 expression. The combined morphologic and immunohistochemical findings were consistent with mesonephric‐like adenocarcinoma.

Figure 3(A, B) H&E stains demonstrating a tumor composed of irregular glands punctuated by foci of microacinar‐to‐solid architecture. (C) PAX 8, positive. (D) TTF1, positive. (E) GATA3, positive. (F) CD10, positive.

**Figure:**
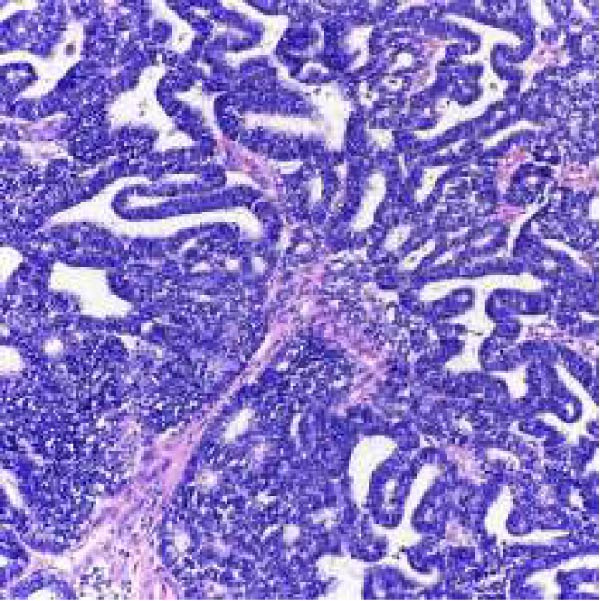
(A)

**Figure:**
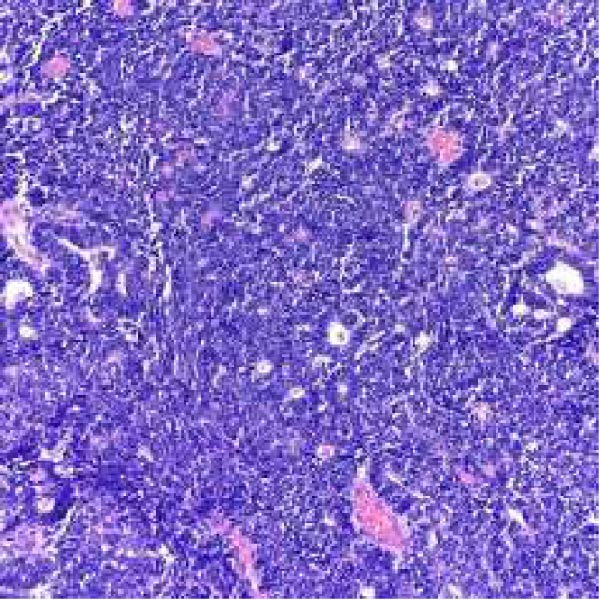
(B)

**Figure:**
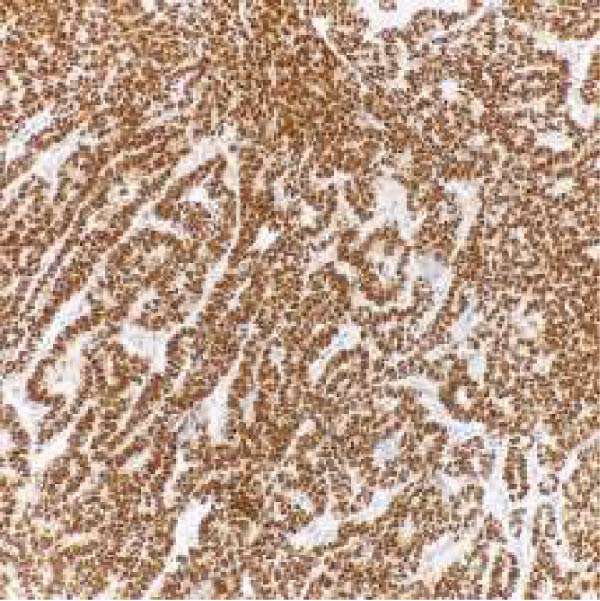
(C)

**Figure:**
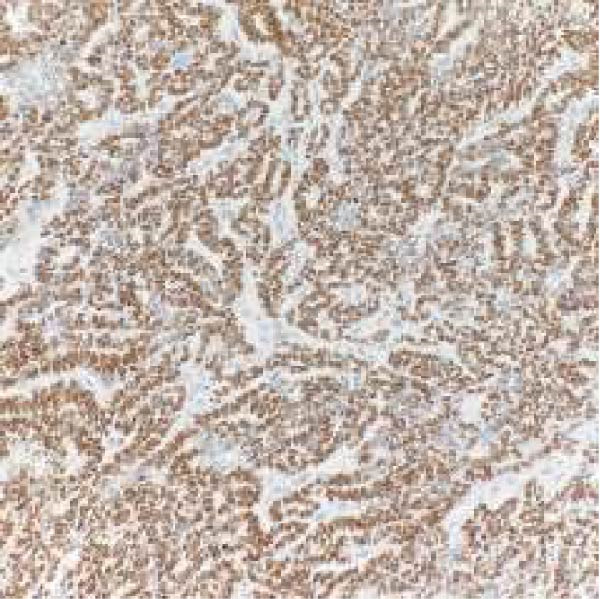
(D)

**Figure:**
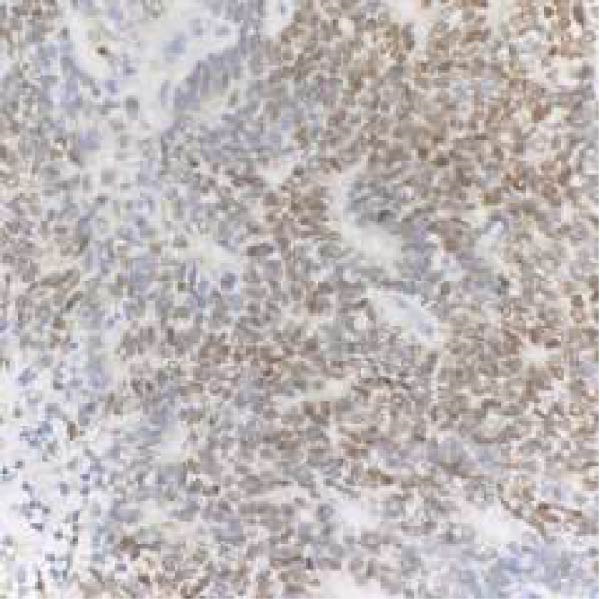
(E)

**Figure:**
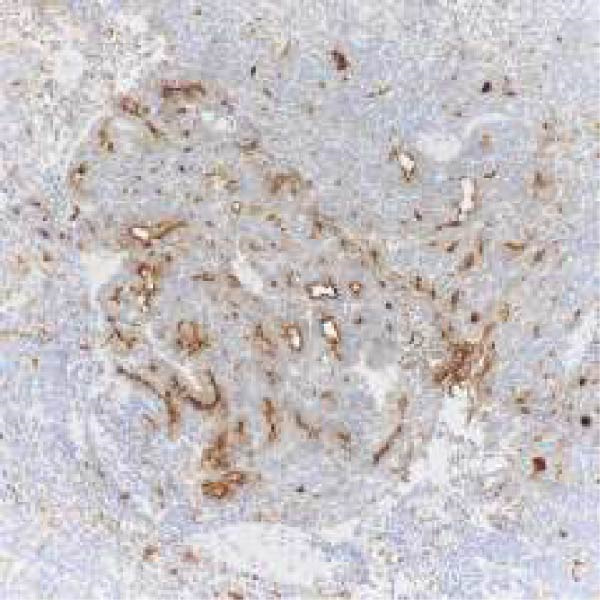
(F)

Molecular profiling revealed a *KRAS* mutation at position G12, typical of mesonephric‐like adenocarcinomas, as well as a mutation in the *CTNNB1* gene. Otherwise, the tumor was HER2 negative, MMR‐proficient, microsatellite stable, and with a low tumor mutational burden (TMB). There was no evidence of TP53 or POLE mutations. Germline genetic testing showed a variant of undetermined significance, CDC73.

Final TN staging using the AJCC 8th edition was pT1a (tumor limited to endometrium or invading less than half the myometrium) and pN0 (no regional lymph node metastasis). FIGO 2023 staging was stage IIC (aggressive histological types with any myometrial involvement).

### 2.2. Treatment Plan

Her postoperative course was unremarkable and she recovered well. She received adjuvant systemic therapy with six cycles of IV carboplatin (AUC 6) and paclitaxel 175 mg/m^2^, which was subsequently dose reduced to 150 mg/m^2^ due to arthralgias and myalgias. She then received adjuvant radiation therapy with external beam radiation therapy to the pelvis (4500 cGy in 25 fractions) followed by high‐dose‐rate vaginal cylinder brachytherapy (1200 cGy in 3 fractions), which she tolerated without any significant side effects.

### 2.3. Follow Up

During surveillance imaging performed in the months following treatment, a 3.7 cm right upper lobe lung mass was identified on CT chest, initially favored to represent a primary lung malignancy. A subsequent endobronchial ultrasound‐guided biopsy confirmed squamous cell carcinoma of the right lung. The patient was evaluated by thoracic surgery and underwent right video‐assisted thoracoscopic surgery (VATS) with right upper lobectomy. Her recovery was uncomplicated. There has been no evidence to suggest metastatic recurrence of the endometrial primary to date.

## 3. Discussion

Mesonephric‐like adenocarcinoma of the uterine corpus is a rare and poorly understood subtype of endometrial cancer. Histologically, mesonephric‐like adenocarcinoma exhibits architectural patterns similar to mesonephric ducts, often displaying tubular or glandular structures lined by cuboidal to columnar epithelial cells [6]. They are therefore often confused with more common adenocarcinomas. In fact, the majority of mesonephric‐like adenocarcinoma of the uterine corpus cases reported in the literature are initially misdiagnosed as low‐grade endometrioid adenocarcinomas and only rarely as high‐grade tumors with clear cell or high‐grade endometrioid features [7]. However, these cells may show immunohistochemical positivity for markers such as GATA3 and TTF1, characteristic of mesonephric differentiation, distinguishing them from more common endometrial adenocarcinomas [6].

Data suggests that mesonephric‐like adenocarcinomas exhibit more aggressive behavior compared to typical endometrial adenocarcinomas, resulting in a poorer prognosis and higher rates of recurrence and metastasis, especially to the lungs [3, 7]. The median progression‐free and overall survival rates for these patients are significantly shorter than those for patients with serous carcinoma, another aggressive subtype [3].

Treatment approaches remain highly variable, often extrapolated from protocols for high‐grade endometrioid or serous carcinomas. Data regarding treatment and prognostic outcomes for patients with mesonephric and mesonephric‐like adenocarcinomas are based largely on published case reports/series and only a few literature reviews. Most patients undergo upfront surgical management which provides optimal diagnostic tissue and some form of local control. In a case series of 40 patients with mesonephric‐like adenocarcinoma of the uterine corpus, almost all patients were treated with upfront surgery, which included hysterectomy and bilateral salpingo‐oophorectomy with or without lymph node dissection [7]. Adjuvant treatment varies widely, however, ranging from radiation therapy, with either vaginal brachytherapy or external beam radiation therapy, to chemotherapy alone, to chemoradiation.

### 3.1. FIGO Restaging and Impact on Treatment

In 2023, FIGO presented an updated staging system for endometrial cancer, incorporating histological subtype and molecular classification to improve prognostic accuracy and guide treatment [8]. This was a dramatic shift from the 2009 FIGO staging system that was largely based on anatomical features. The consideration of high‐risk histologies and other prognostic variables such as lymphovascular invasion (LVSI) in the new staging system has led to the upstaging of many cancers [9–11]. Based on the FIGO 2023 staging, mesonephric‐like carcinoma is considered a high‐risk histology [8]. For the patient presented in the case study, according to the 2009 FIGO system, the tumor would be stage IA due to its myometrial invasion, but based on the new staging system, it is upstaged to IIC due to the high‐risk histology. Notably, the upstaging of this tumor from FIGO stage IA under the 2009 criteria to stage IIC under the 2023 system highlights the importance of recognizing aggressive histologies, as stage IIC disease is associated with a significantly poorer overall survival compared to earlier‐stage disease [12]. Other aggressive histological types identified by the FIGO 2023 restaging include serous adenocarcinomas, clear cell adenocarcinomas, high‐grade endometrioid endometrial carcinoma, gastrointestinal‐type mucinous endometrial carcinoma, undifferentiated carcinomas, and carcinosarcomas [8]. Because these subtypes are associated with higher risks of recurrence and distant metastasis, they are generally managed with intensified multimodality therapy, including comprehensive surgical staging followed by adjuvant systemic therapy, radiation therapy, or a combination of both, depending on stage, histologic subtype, and additional risk factors [13, 14]. Increasingly, targeted therapies or immunotherapy are being integrated into management based on molecular profiling [14].

The 2023 FIGO update also incorporated molecular classification. This change is based on findings from the integrated genomic characterization of endometrial carcinoma by the landmark genomics program, The Cancer Genome Atlas (TCGA) [15]. Based on this research, most endometrial cancers fall into (1) POLE (ultramutated) (2), MSI (hypermutated), (3) copy‐number low (endometrioid), and (4) copy‐number high (serous‐like). This classification is further supported by recent studies, including the Proactive Molecular Risk Classifier for Endometrial Cancer (ProMisE) [16]. For the patient presented in the case study, molecular testing of the tumor revealed MMR proficiency, POLE nonmutant and P53 normal type, a molecular subtype of intermediate prognosis [17].

Despite the excitement surrounding the FIGO 2023 staging and its value in including prognostic factors, further validation is warranted. Moreover, the trials guiding current practice and treatment planning are based on old staging systems, and there are no widely adopted treatment guidelines based on the new staging [18]. Studies on the horizon, such as PORTEC‐4, are integrating molecular profiling into treatment management [19]. Preliminary results from PORTEC‐4 demonstrate that a molecular profiling approach can help identify patients who can safely avoid adjuvant therapy but also ensure those at higher risk receive more appropriate, intensified treatment [20]. This marks a shift toward more personalized approaches in gynecologic oncology [19].

## 4. Conclusion

Although clinical outcomes data for mesonephric‐like adenocarcinoma are limited due to its rarity and likelihood of misclassification, it is generally considered a more aggressive subtype of endometrial cancer. In the presented case, the updated 2023 FIGO staging system upstaged this patient from stage IA to IIC due to her aggressive histology, illustrating the clinical implications of the FIGO staging update.

The FIGO update promises to offer a more nuanced understanding of endometrial cancer and better prognostic performance. Preliminary data from PORTEC‐4 confirm its potential to stratify patients more accurately for adjuvant therapy. However, changes in staging must be matched by updates in treatment guidelines, especially for rare and aggressive histologies like mesonephric‐like adenocarcinoma, which currently lacks a standardized treatment protocol. Further research is needed to define an optimal treatment protocol in this rare subtype. A personalized, multidisciplinary treatment approach remains crucial for optimizing outcomes in affected patients [3, 7, 21].

## Author Contributions

Conceptualization: Joy Ogunmuyiwa and Judy Hayek. Writing – original draft preparation: Joy Ogunmuyiwa, Judy Hayek, Alexandra Hamilton, and Daniel Levitan. Writing – review and editing: Joy Ogunmuyiwa, Judy Hayek, Alexandra Hamilton, Jocelyn Ogunmuyiwa, Daniel Levitan, Hani Ashamalla, and Constantine Gorelick.

## Funding

The authors have nothing to report.

## Disclosure

All authors have read and agreed to the published version of the manuscript.

## Consent

Written informed consent was obtained from the patient for publication of this case report and accompanying images.

## Conflicts of Interest

The authors declare no conflicts of interest.

## Data Availability

The data that support the findings of this study are available upon request from the corresponding author. The data are not publicly available due to privacy or ethical restrictions.
